# Identification and expression profiling of *SmGATA* genes family involved in response to light and phytohormones in eggplant

**DOI:** 10.3389/fpls.2024.1415921

**Published:** 2024-05-28

**Authors:** Yanyan Wang, Xinyun Li, Yunrong Mo, Caiqian Jiang, Ying Zhou, Jingyi Hu, Youling Zhang, Junheng Lv, Kai Zhao, Zhenya Lu

**Affiliations:** ^1^ Key Laboratory of Vegetable Biology of Yunnan Province, College of Landscape and Horticulture, Yunnan Agricultural University, Kunming, China; ^2^ College of Resources and Environmental Sciences, National Academy of Agriculture Green Development, China Agricultural University, Beijing, China

**Keywords:** *Solanum melongena* L., GATA, gene family, light, hormones

## Abstract

GATA proteins are transcription factors of zinc finger proteins, which play an important role in plant growth development and abiotic stress. However, there have been no identification or systematic studies of the *GATA* gene family in eggplant. In this study, 28 *SmGATA* genes were identified in the genome database of eggplant, which could be divided into four subgroups. Plant development, hormones, and stress-related *cis*-acting elements were identified in promoter regions of the *SmGATA* gene family. RT-qPCR indicated that 4 *SmGATA* genes displayed upregulated expressions during fruit developmental stage, whereas 2 *SmGATA* genes were down-regulated expression patterns. It was also demonstrated that *SmGATA* genes may be involved in light signals to regulate fruit anthocyanin biosynthesis. Furthermore, the expression patterns of *SmGATA* genes under ABA, GA and MeJA treatments showed that the *SmGATA*s were involved in the process of fruit ripening. Notably, *SmGATA4* and *SmGATA23* were highly correlated with the expression of anthocyanin biosynthesis genes, light-responsive genes, and genes that function in multiple hormone signaling pathways and the proteins they encoded were localized in the nucleus. All these results showed *GATA* genes likely play a major role in regulating fruit anthocyanin biosynthesis by integrating the light, ABA, GA and MeJA signaling pathways and provided references for further research on fruit quality in eggplant.

## 1 Introduction

Transcription factors (TFs) play a critical role in regulating gene expression in plants. They are specialized proteins capable of interacting with specific DNA sequences, usually within a gene’s promoter region, to regulate its transcription into messenger RNA (mRNA) and subsequently into proteins (Riechmann et al., 2000; Fan et al., 2021). Furthermore, TFs can form complexes with other transcription factors, coactivators, and corepressors to finely regulate gene expression. These complexes allow for the integration of multiple signals and contribute to the precise control of transcriptional activity (Xu, 2020; Lai et al., 2022). Besides their involvement in transcriptional regulation, transcription factors also participate in various physiological and biochemical pathways during the development of higher plants, including plant growth and development (Strader et al., 2022), metabolic networks (Rueda-López et al., 2015), stress responses (Yao et al., 2022), and disease resistance (Li et al., 2017). Within this category, the GATA family has emerged as a prominent group of transcription factors, widely participating in key mechanisms related to plant growth, development, and stress response.

GATA proteins, found across eukaryotes including animals, plants, and fungi, are evolutionarily conserved regulatory factors. They derive their name from their distinctive capability to recognize and bind to the T/AGATAA/G core sequences, exerting regulatory control over various biological processes (Lowry and Atchley, 2000; Behringer and Schwechheimer, 2015). GATA transcription factors typically contain one or two highly conserved type IV zinc finger structural modules, characterized by the sequence signature C-X_2_-CX_17-20_-C-X_2_-C (C, cysteine; X, other residues), followed by a highly basic region (Schwechheimer et al., 2022). The zinc finger structure represents a prevalent DNA-binding domain, stabilizing its three-dimensional conformation through coordination bonds between zinc ions and specific amino acid residues within the protein (Takatsuji, 1998). This structural module in GATA transcription factors enables precise recognition and binding to the promoter regions of target genes, thus regulating their transcriptional activity. The initial discovery of a plant GATA factor, Ntl1 (NIT2-like), from Nicotiana tabacum, is pivotal for understanding nitrogen metabolism (Daniel-Vedele and Caboche, 1993). Presently, GATA TFs have been detected in numerous plant species, such as *Arabidopsis thaliana* (30 members) (Reyes et al., 2004), *Oryza sativa* (28 members) (Reyes et al., 2004), *Solanum lycopersicum* (30 members) (Yuan et al., 2018), *Malus domestica* (35 members) (Chen et al., 2017), *Arachis hypogaea* (45 members) (Li et al., 2023), *Solanum tuberosum* (49 members) (Yu et al., 2021), and *Triticum aestivum* (79 members) (Feng et al., 2022).

GATA transcription factors, key regulators in plants, have diverse biological functions, such as regulating light responses (Jeong and Shih, 2003), chlorophyll synthesis (An et al., 2014), and responses to environmental stress (Richter et al., 2013). By recognizing and binding to specific DNA sequences, these transcription factors regulate the expression of relevant genes, thereby influencing plant growth, development, and adaptability. Regarding light response regulation, GATA transcription factors modulate plant adaptive responses to changes in light exposure. For instance, GATA2 in *Arabidopsis thaliana* mediates photomorphogenesis and plays a pivotal role in light signal transduction (Luo et al., 2010). Furthermore, two *GATA* genes in *A. thaliana* were strongly light-regulated and expressed in photosynthetic organs. Several *GATA* genes have shown responsiveness to light, darkness, and circadian rhythm changes (Manfield et al., 2007). Another example is PdGATA19/PdGNC from poplar (*Populus deltoides*), which contributes to photosynthesis and growth (An et al., 2020). Additionally, *GATA* genes have been found to be extensively involved in hormone signal transduction. In *A. thaliana*, *AtGATA12* expression is downregulated by gibberellin (GA), promoting seed dormancy (Ravindran et al., 2017), while GATA2 mediates cross-talk between the brassinosteroid and light signaling pathways, regulating plant photomorphogenesis (Luo et al., 2010). PdGNC can be significantly induced by abscisic acid (ABA) and dehydration, thereby improving water use efficiency by reducing stomatal aperture and enhancing water deficit tolerance in poplar (Shen et al., 2021). Proteomic analysis findings indicate that GATAs can interact with JAZ, a key regulator in the jasmonic acid (JA) signaling pathway (Sen et al., 2016).

Eggplant (*Solanum melongena* L.), a significant economic crop, is cultivated globally for its nutrient-rich fruits. The fruits of purple eggplant have a high content of anthocyanins, which is highly beneficial for health (Bor et al., 2006; García et al., 2008). So far, serval TFs including *AP2*/*ERF* and *WRKY* gene family under abiotic stress conditions from eggplant have been well-characterized (Yang et al., 2020; Li et al., 2021a). However, systematic studies on the eggplant GATA TFs superfamily have not been attempted so far. In the present investigation, we screened 28 *GATA* genes from the eggplant genome and investigated their phylogenetic correlation, gene structure and motif pattern. We further analyzed their chromosome distribution and putative cis-acting elements on the promoters. More importantly, we studied the expression patterns of *SmGATA* genes during fruit development. In addition, we analyzed the expression patterns of *GATA* genes in response to light and multiple hormone signals. The results of this study will provide a platform and cues for further studies related to the functional characterization of eggplant GATAs and identification of pivotal GATA involved in growth and development.

## 2 Materials and methods

### 2.1 Plant growth conditions and hormone treatments

The eggplant cultivar ‘zaohongqie 2’ was used as a material, which were cultivated in a climate chamber at a circadian temperature of 22–25°C with a 16 h photoperiod. Fruit samples were collected at 6 time points during the growing season, including 5 days after flowering (5 d), 10 d, 15 d, 20 d 25 d and 30 d, and which were used to study the expression characteristics of *SmGATA* genes.

A fruit bagging experiment was conducted at 0 DAF. The bags were removed from eggplant fruits at 25 days after flowering (25 DAF). Samples were taken at 0 d, 2 d, 4 d, 6 d, and 8 d after bag removal. For hormone treatment, Similarly, the eggplant fruit that had been bagged for 25 d will be removed from the bag and then which were treated with water (control), 200 μM ABA, 200 μM GA and 200 μM MeJA. Three replicates were performed in the experiment, with 3 fruits per replicate. The fruits were immediately frozen in liquid nitrogen and stored at -80°C.

### 2.2 Identification of *GATA* genes in the eggplant genome

The deduced amino acid sequences of 30 *GATA* genes in *Arabidopsis thaliana* were used as query sequences for a BlastP search of the eggplant genome database (http://www.eggplant-hq.cn/Eggplant/home/index). The CDD database (https://www.ncbi.nlm.nih.gov/Structure/cdd/cdd.shtml) and the SMART database (http://smart.embl-heidelberg.de) were used to analyze the domains of the candidate GATA proteins. The molecular weights, isoelectric points (pIs), and grand average of hydropathicity (GRAVY) values of the SmGATA proteins were calculated using the ExPASy website (https://web.expasy.org/protparam/).

### 2.3 Phylogenetic analysis and gene structure analysis

Multiple sequence alignments of GATA proteins were analyzed using ClustalW in BioEdit (http://bioedit.software.informer.com), and the phylogenetic tree was constructed with the neighbor-joining algorithm in MEGA 6.0 (Tamura et al., 2013). Bootstrap analysis was carried out with 1000 replicates. WebLogo (http://weblogo.berkeley.edu/logo.cgi) was used to generate sequence logos of the conserved domains. Furthermore, MEME (http://meme-suite.org/) was used tosearch for motifs in all *GATA* genes. A structure analysis of the *SmGATA* genes was performed using the Gene Structure Display Server (GSDS) (http://gsds.gao-lab.org/).

### 2.4 *Cis*-acting element prediction and chromosome duplication events analysis

The promoter sequences (2 kb upstream from ATG) were extracted from eggplant whole genome, and *cis*-elements in the promoters were predicted using the PlantCARE online program (http://bioinformatics.psb.ugent.be/webtools/plantcare/html/). TBtools v1.106. https://github.com/CJ-Chen/TBtools/releases was used to map the genes to chromosome duplication events analysis (Chen et al., 2022).

### 2.5 RT-qPCR analysis

Total RNA was extracted from the samples using a Plant Total RNA Extraction kit (Huayueyang Biotechnology, Beijing, China). The integrity of the RNA was examined by 1.0% agarose gel electrophoresis, and cDNA was synthesized from the RNA using a Reverse Transcription kit (TaKaRa Biotechnology, Dalian, China). All primers used for RT-qPCR are listed in **Supplementary Table S1**. RT-qPCR was performed using SYBR Premix Ex Taq (Yeasen Biotechnology (Shanghai) Co., Ltd) with the ABI 7500 System. All experiments were carried out with three biological replicates. The relative expression value of each gene was quantified using the 2^-△△Ct^ method (Livak and Schmittgen, 2001).

### 2.6 Co-expression network construction and visualization

The differentially expressed genes in the anthocyanin biosynthesis pathway, light signaling pathway, and hormone signaling-related pathways were identified in eggplant fruits with different colors (**Supplementary Table S2**). Pearson’s correlation tests were performed with SPSS v25 software using the FPKM values of both purple eggplant ‘YN-1’ and yellow eggplant ‘S-12’ samples. Any two genes with an absolute Pearson correlation coefficient of ≥ 0.9 and a *p*-value of ≤ 0.05 were considered to be significantly co-expressed genes. The co-expression network was visualized using Cytoscape v3.5.1 software.

### 2.7 Subcellular localization analysis

To examine the subcellular locations of the SmGATA proteins, two full-length *S*mGATA open reading frames (ORFs) (for SmGATA4 and SmGATA23) without the stop codon were amplified from cDNA from eggplant fruit. Each amplification product was cloned into the pCAMBIA1302 vector with green fluorescence protein (GFP) label under the control of the *CaMV35S* promoter. The gene-specific primers are listed in **Supplementary Table S3**.

*Nicotiana benthamiana* plants were grown in a plant growth chamber at 26°C under a 16-hr light/8-hr dark regimen until they were approximately 15 cm tall and infiltrated with *Agrobacterium* strain EHA105 harboring the constructs described above. Infiltration was performed as described by (Sparkes et al., 2006). The agroinfiltrated leaves were photographed 2 days after infiltration. GFP fluorescence images were captured using an Olympus laser-scanning confocal microscope.

### 2.8 Data analysis

All data were analyzed using Student’s *t*-test with the software SPSS 25.0. The values are represented as the means ± SD. *P* < 0.05 was considered as statistically significant.

## 3 Result

### 3.1 Identification of *SmGATA* genes in Solanum melongena

To identify and obtain the *GATA* genes in the eggplant genome, the *Arabidopsis* GATA proteins were used as a query to search against the eggplant genome via BLAST. Then, the results of the blast search through confirmation of the presence of the GATA domain using SMART. After removing the redundant sequences, 28 full-length *GATA* homologous sequences were identified in the genomes of eggplant (**Supplementary Figure S1**). The detailed information of *SmGATA* genes were listed in **Table 1**, including gene name, protein length, chromosome location, molecular weight, theoretical isoelectric point, aliphatic index, and GRAVY. The 28 SmGATA proteins had diverse molecular length and weight, ranging from 117 (SmGATA23) to 542 (SmGATA20) in amino acid length. SmGATA23 showed the lowest value of the molecular weight (13.24 kDa), while the highest of the molecular weight (59.98 kDa) was observed in SmGATA22. Theoretical isoelectric points of these SmGATA proteins varied from 5.15 (SmGATA2) to 9.87 (SmGATA3) and the value of the aliphatic index ranged from 41.12 (SmGATA9) to 71.76 (SmGATA3). The GRAVY of all SmGATAs were less than zero, indicating the hydrophilic nature of SmGATA proteins (**Table 1**).

**Table 1:** The information on the *GATA* gene family in *Solanum melongena* L.

Gene Name	Accession number	Protein/AA	Chrom	Chr start	Chr end	MW (Da)	pI	Aliphatic index	GRAVY
*SmGATA1*	Smechr0100184.1	326	Chr1	1591223	1592866	36007.44	6.46	52.58	-0.611
*SmGATA2*	Smechr0100542.1	381	Chr1	4819785	4825865	41287.78	5.15	59.92	-0.640
*SmGATA3*	Smechr0100954.1	148	Chr1	9085976	9087125	16066.85	9.87	71.76	-0.603
*SmGATA4*	Smechr0101674.1	247	Chr1	16634196	16635510	27860.50	6.45	49.35	-0.913
*SmGATA5*	Smechr0102738.1	140	Chr1	41361542	41362611	14928.08	9.51	58.57	-0.640
*SmGATA6*	Smechr0200677.1	253	Chr2	45893818	45895037	28132.46	7.01	57.39	-0.861
*SmGATA7*	Smechr0200741.1	211	Chr2	47448341	47449594	23599.68	7.07	38.48	-0.944
*SmGATA8*	Smechr0202290.1	332	Chr2	67474700	67476503	35717.69	5.69	56.08	-0.662
*SmGATA9*	Smechr0202357.1	240	Chr2	68035466	68036702	26564.38	8.73	41.12	-0.757
*SmGATA10*	Smechr0300289.1	303	Chr3	3353845	3355048	33778.78	8.47	51.45	-0.852
*SmGATA11*	Smechr0303516.1	355	Chr3	94689658	94691529	38492.03	5.77	57.75	-0.671
*SmGATA12*	Smechr0400753.1	243	Chr4	17172444	17173370	27736.58	7.10	51.32	-0.930
*SmGATA13*	Smechr0402001.1	356	Chr4	73888628	73894111	38875.44	5.30	70.62	-0.607
*SmGATA14*	Smechr0500398.1	269	Chr5	4605066	4608557	30527.34	8.11	68.07	-0.566
*SmGATA15*	Smechr0500497.1	196	Chr5	5722265	5725332	21147.14	9.78	58.72	-0.724
*SmGATA16*	Smechr0500708.1	328	Chr5	7981703	7986086	35903.10	5.94	61.22	-0.623
*SmGATA17*	Smechr0502694.1	168	Chr5	80598117	80601599	18366.64	9.90	53.45	-0.933
*SmGATA18*	Smechr0601814.1	536	Chr6	76462233	76469689	59632.97	7.58	70.73	-0.664
*SmGATA19*	Smechr0602825.1	212	Chr6	86599722	86600526	24053.05	8.38	52.45	-0.797
*SmGATA20*	Smechr0800034.1	542	Chr8	607183	612746	60373.55	7.86	66.35	-0.708
*SmGATA21*	Smechr0801395.1	357	Chr8	66986313	66987708	40067.54	6.31	56.81	-0.763
*SmGATA22*	Smechr0802078.1	537	Chr8	81357736	81363510	59976.08	6.71	67.67	-0.702
*SmGATA23*	Smechr0902133.1	117	Chr9	82598683	82599668	13238.67	9.67	70.94	-0.686
*SmGATA24*	Smechr0902475.1	278	Chr9	86977346	86980304	30801.73	8.29	66.98	-0.632
*SmGATA25*	Smechr1102522.1	336	Chr11	97906524	97911222	36931.35	6.02	55.98	-0.662
*SmGATA26*	Smechr1200166.1	311	Chr12	2240979	2243863	34639.57	9.57	54.60	-0.833
*SmGATA27*	Smechr1200608.1	326	Chr12	12987604	13006304	34426.80	5.58	62.27	-0.624
*SmGATA28*	Smechr1201886.1	327	Chr12	73264242	73269660	35851.98	8.75	68.23	-0.496

AA, amino acid residues; Chrom, chromosome; MW, molecular weight; pI, theoretical isoelectric point; GRAVY, grand average of hydropathicity.

### 3.2 Characteristics and phylogenetic analysis of the *SmGATA* gene family

In order to clarify the characteristics of GATA proteins in eggplant, we first extracted their conserved domain sequences and visualized them (**Supplementary Figure S2**). Similar to what has been found in other plant species, the conserved domain of GATA proteins in eggplant also contained a consensus sequence of C-X_2_-C-X_18_/_20_-C-X_2_-C. It is worth noting that besides the highly conserved cysteine residues, there are also many highly conserved amino acid residues in this domain, which may be related to the functions of GATA proteins in eggplant.

**Figure 1:**
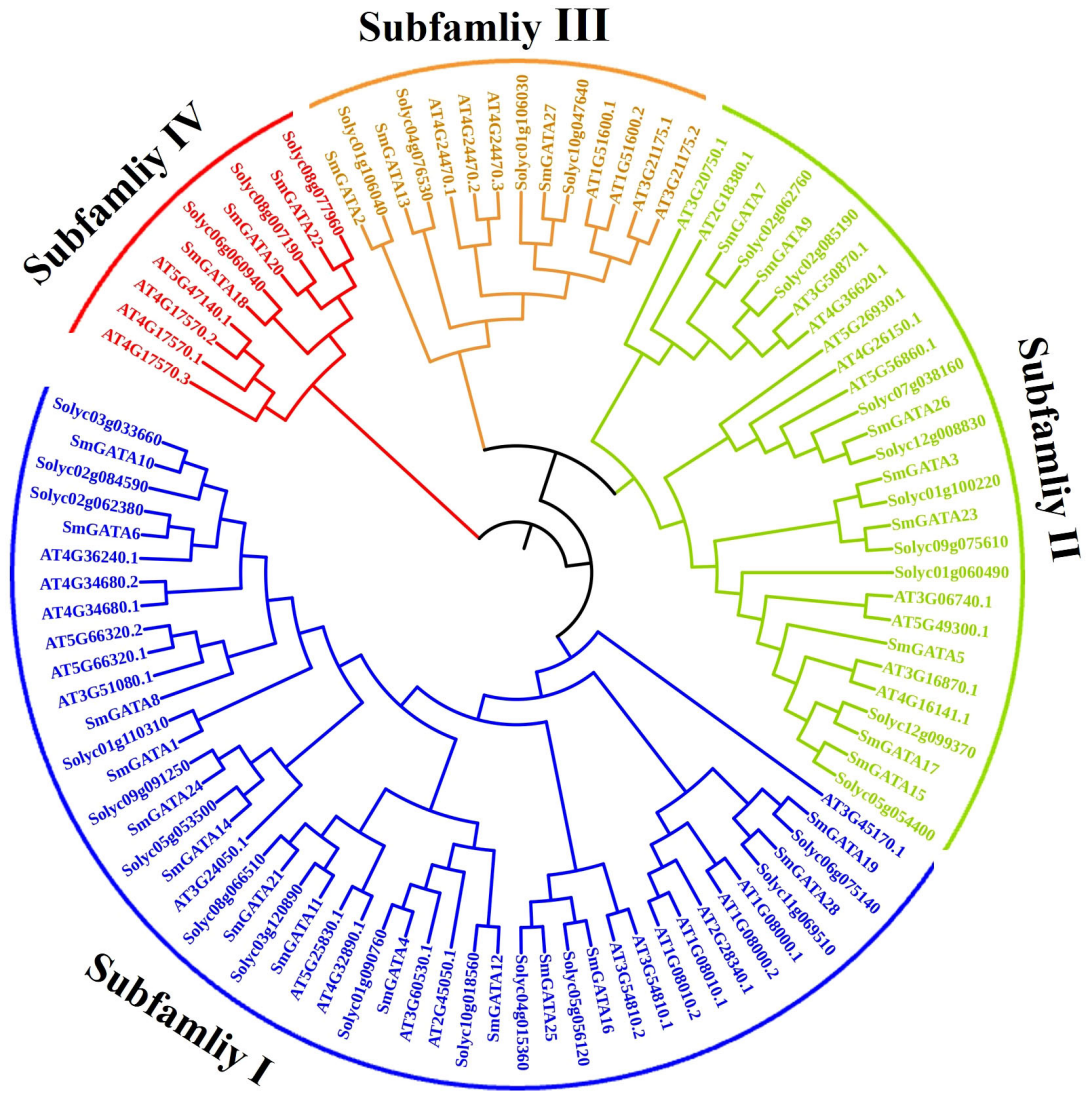
Phylogenetic analysis of GATA proteins in eggplant, tomato and Arabidopsis.

**Figure 2:**
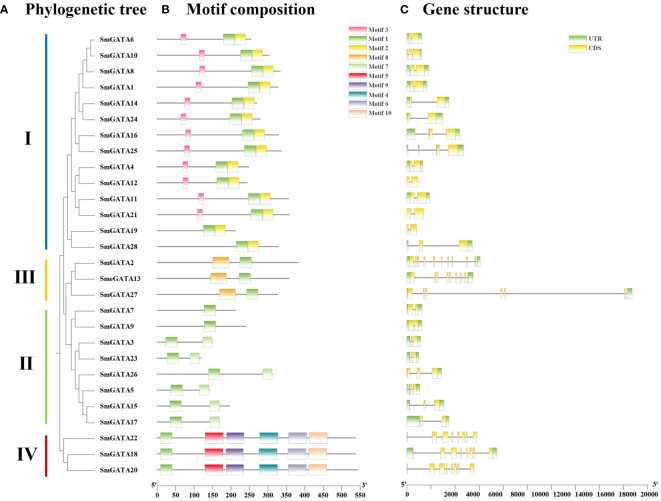
Phylogenetic tree, motif composition and gene structure of GATA genes in eggplant. **(A)** The phylogenetic tree is constructed by the full-length sequences of eggplant GATA proteins with 1000 replicates on each node. **(B)** The amino acid motifs (numbered 1-10) in SmGATAs are displayed in ten colored boxes, and black lines indicate protein sequence length. **(C)** Green rectangles, yellow rectangles lines represent the UTR (untranslated region), CDS (coding sequence or exons), respectively.

To explore the evolutionary relationship and functional divergence of the SmGATA members, a phylogenetic tree was constructed with MEGA6.0 by using the neighbor-joining method according to SmGATAs, AtGATAs and SlGATAs protein sequences (**Figure 1**), respectively. All SmGATA proteins were clustered into four major subfamilies. Among them, subfamily I contained the largest SmGATA members (14/28), followed by subfamily II (8/28),subfamily III (3/28), and then subfamily IV (3/28) (**Figure 2A**).

### 3.3 Structures and conserved motifs analysis of SmGATA family

To explore the gene and protein sequence structures of eggplant GATA family members, visualization of their motif compositions and intron/exon structures was performed. Analysis of the motif composition of SmGATAs identified a total of 10 conserved motifs (**Figure 2B**). Consistent with the expected results, all proteins contained motif 1, annotated as the GATA domain. In subfamily I, most proteins had three motifs (12/14); in subfamily II, the number of motifs was relatively small, with most proteins having two motifs (6/8) and the remaining two proteins only have one motif; in subfamily III, all three SmGATAs had two motifs, and motif 1 was positioned after motif 8 in the amino acid sequence. Furthermore, in subfamily IV, The number of motifs was the highest, with all three SmGATAs had 6 motifs. The differences in the number and variety of conserved motifs in SmGATA proteins reflected the structural diversity of these proteins, while predicting that they have different biological functions.

Based on the eggplant genome sequence, a gene structure map of Solanum melongena GATAs was constructed (**Figure 2C**). 28 SmGATAs all contain no less than 2 CDS regions. Among them, subfamilies I and II both contained 2-4 CDSs, while SmGATA2 in subfamily III had the 10 CDS segments, which was the most abundant. For the untranslated region (UTR), SmGATA12, 19, 20, 21 and 22 had no UTRs, and the remaining GATAs had their UTRs distributed almost at both ends of the gene.

### 3.4 Analysis of *cis*-acting elements

**Figure 3:**
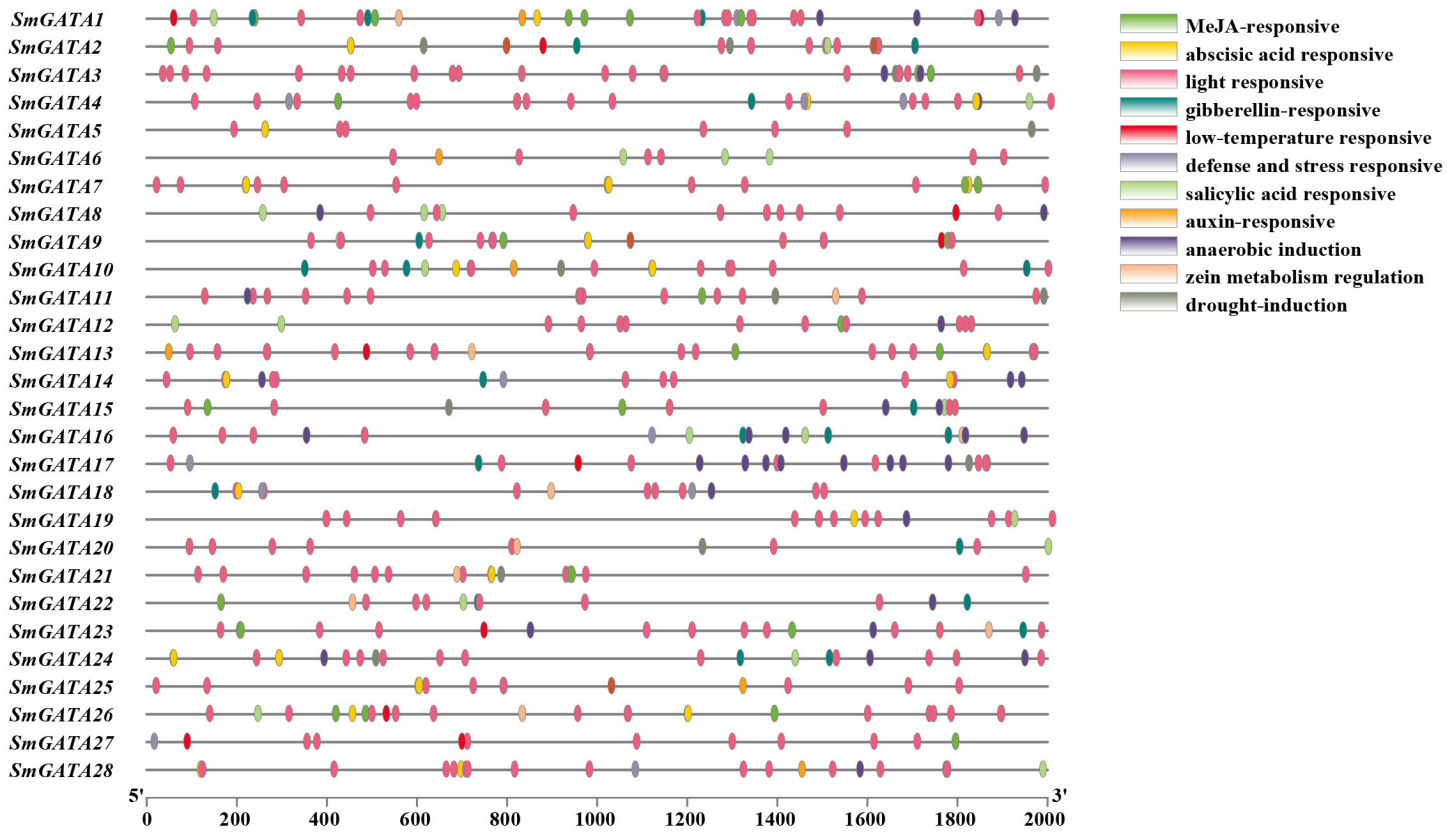
Schematic representations of the predicted regulatory elements in the *SmGATA* family.

*Cis*-elements are involved in gene regulation through the interaction with their corresponding trans-regulators and the study of the proposed cis-elements will provide valuable information for the expression of eggplant *GATA* genes. Therefore, the promoter region of *GATA* genes in eggplant were obtained and taken to Plantcare database for prediction (**Figure 3**). The results showed that a total of 26 elements were predicted. among which CAAT-box and TATA-box were present in all *SmGATA* genes. The remaining elements included light responsive elements, defense response elements and hormone induction elements, such as those for abscisic acid (ABA), auxin (IAA), gibberellin (GA), Salicylic acid (SA) and Methyl jasmonate (MeJA) elements.

### 3.5 Chromosome duplication events analysis of SmGATAs

**Figure 4:**
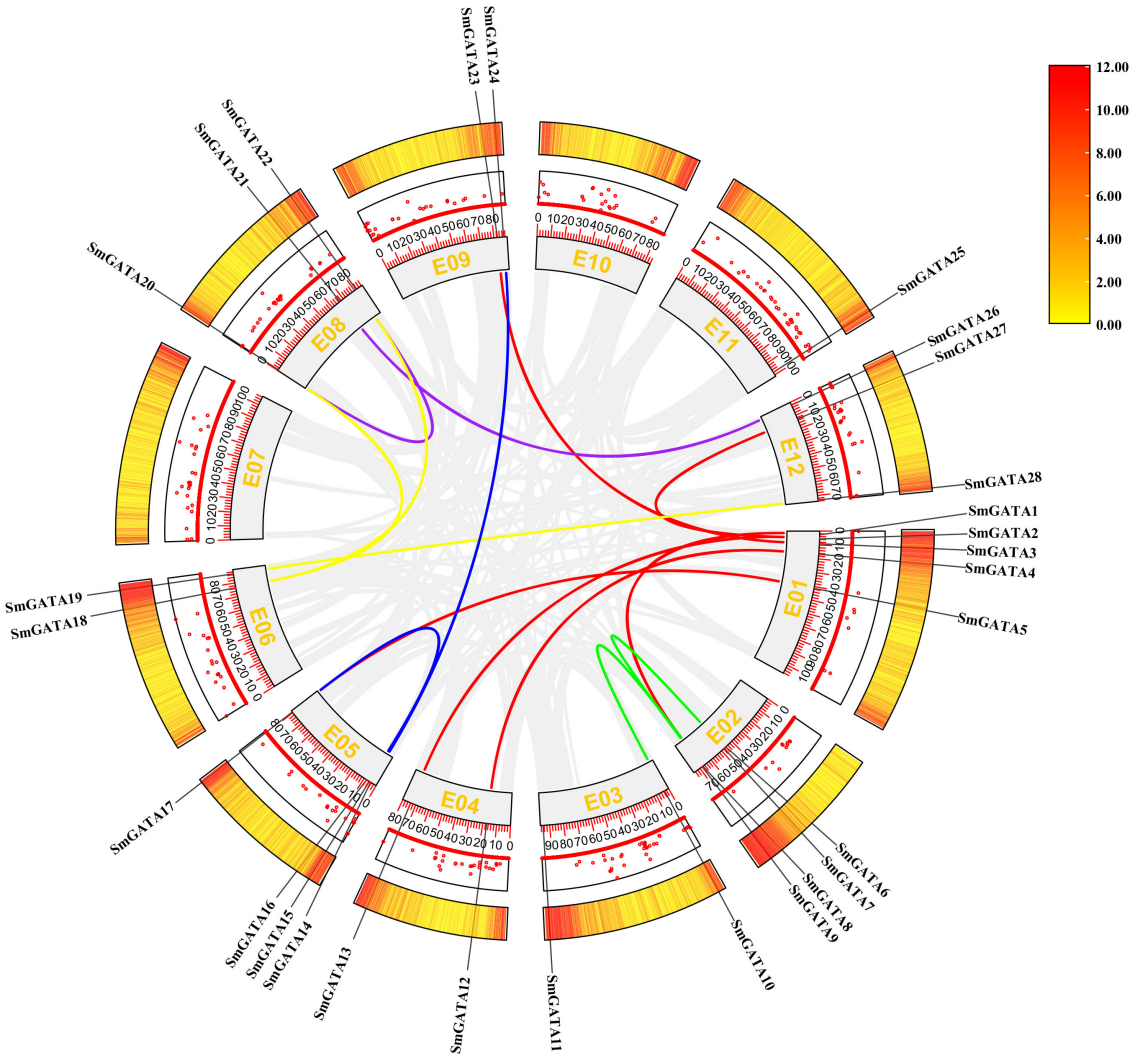
Collinearity of *GATA* genes in eggplant. Colored lines indicate duplicated *GATA* gene pairs. The chromosome number is shown inside each chromosome.

In this study, we analyzed gene duplication events of the 28 *SmGATA* genes to further explore the evolutionary relationships among the *GATA* genes (**Figure 4**) and identified 15 pairs of quasi-homologous GATAs in the eggplant genome originated from duplication, of which five pairs belonged to subfamily I. SmGATAs were unevenly distributed among the 12 linked regions (EG) of the eggplant genome, with EG1 containing the largest number of SmGATAs (6/15), followed by EG6 containing three SmGATAs (3/15), while EG7, EG10 and EG11 had no duplicated genes.

### 3.6 Expression patterns of *SmGATA* genes during eggplant fruit developmental and ripening

**Figure 5:**
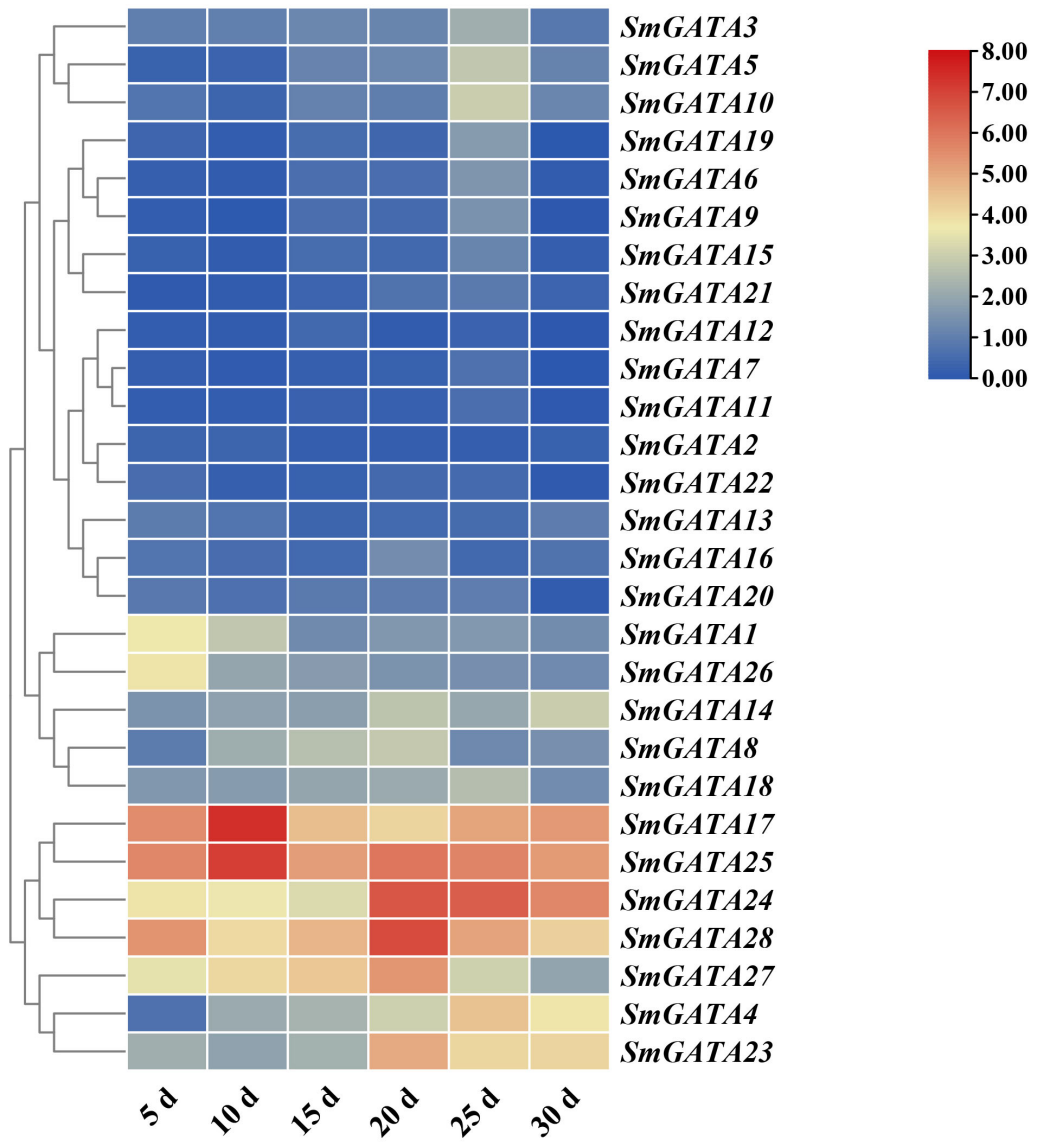
Expression profiles of the eggplant *SmGATA* genes during fruit developmental.

GATAs are a class of transcription factors closely related to growth and development (Jeong and Shih, 2003; Richter et al., 2013; An et al., 2014). Therefore, we analyzed the role of *SmGATA* genes in eggplant fruit development and maturation (**Figure 5**), the expression patterns of 28 *SmGATA* genes in five developmental stages of eggplant fruit were studied. Different members of the *SmGATA* genes showed distinct expression patterns during different eggplant fruit developmental stages. As shown in **Figure 5**, three genes (*SmGATA4*, *23* and *24*) displayed upregulated expressions during fruit developmental stage and the expression level of *SmGATA27* showed a trend of first increasing and then decreasing. Two genes (*SmGATA1* and *SmGATA26*) were down-regulated expression patterns. These results indicated that some *SmGATA* genes maybe play multiple important roles in eggplant fruit development.

### 3.7 Regulation of the expression of *SmGATA* genes family during light induction

**Figure 6:**
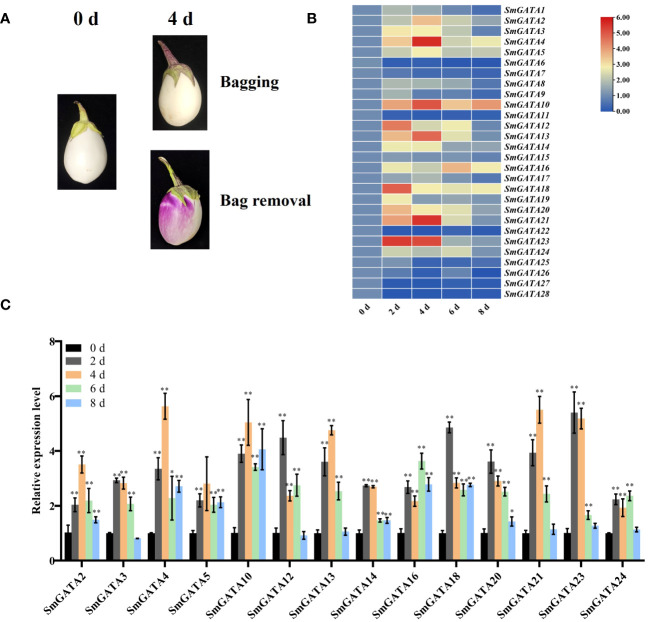
Expression profiles of *SmGATA* genes in ‘Zaohongqie 2’ fruit following light induction via bag removal. **(A)** Colour changes of ‘Zaohongqie 2’ fruits after bag removal. **(B)** Hierarchical clustering of the expression profiles of 28 *SmGATA* genes after bag removing. **(C)** RT-qPCR analysis of 14 selected *SmGATA* genes following light induction. *SmActin* was used as the internal reference control to normalize template levels. The relative mRNA levels are represented as the mean ± SD (n=3). Statistically significant differences were assessed using Student’s *t*-test (*P < 0.05, **P < 0.01).

The classification of anthocyanins in eggplants into photosensitive and non-photosensitive types is based on how these pigments respond to light exposure during the growth and development of the eggplant fruit (He et al., 2019; Ohyama et al., 2022), and the signaling pathways for their synthesis are unclear. GATA motif as a light-response promoter element, is usually the binding site of GATA transcription factors (Luo et al., 2010). In order to analyze the potential role of *SmGATA* gene in photosensitive eggplants in the biosynthesis of anthocyanins, we placed bags on developing eggplant fruits at 0 DAF to block their exposure to light and removed the bags at 25 DAF. The fruits rapidly changed color after bag removal (**Figure 6A**). We used RT-qPCR to measure *SmGATA* genes expression in eggplant after bag removal. Different genes showed different expression levels (**Figure 6B**). As shown in **Figure 6C**, 14 *SmGATA* genes were upregulated more than two-fold after light induction. In particular, *SmGATA4*, *13*, *21* and *23* were upregulated approximately 5-fold at 4 d of light induction. These results represented that these *GATA* genes function in light-induced anthocyanin biosynthesis in eggplant.

### 3.8 Regulation of the expression of *SmGATA* genes family during hormones treatment

**Figure 7:**
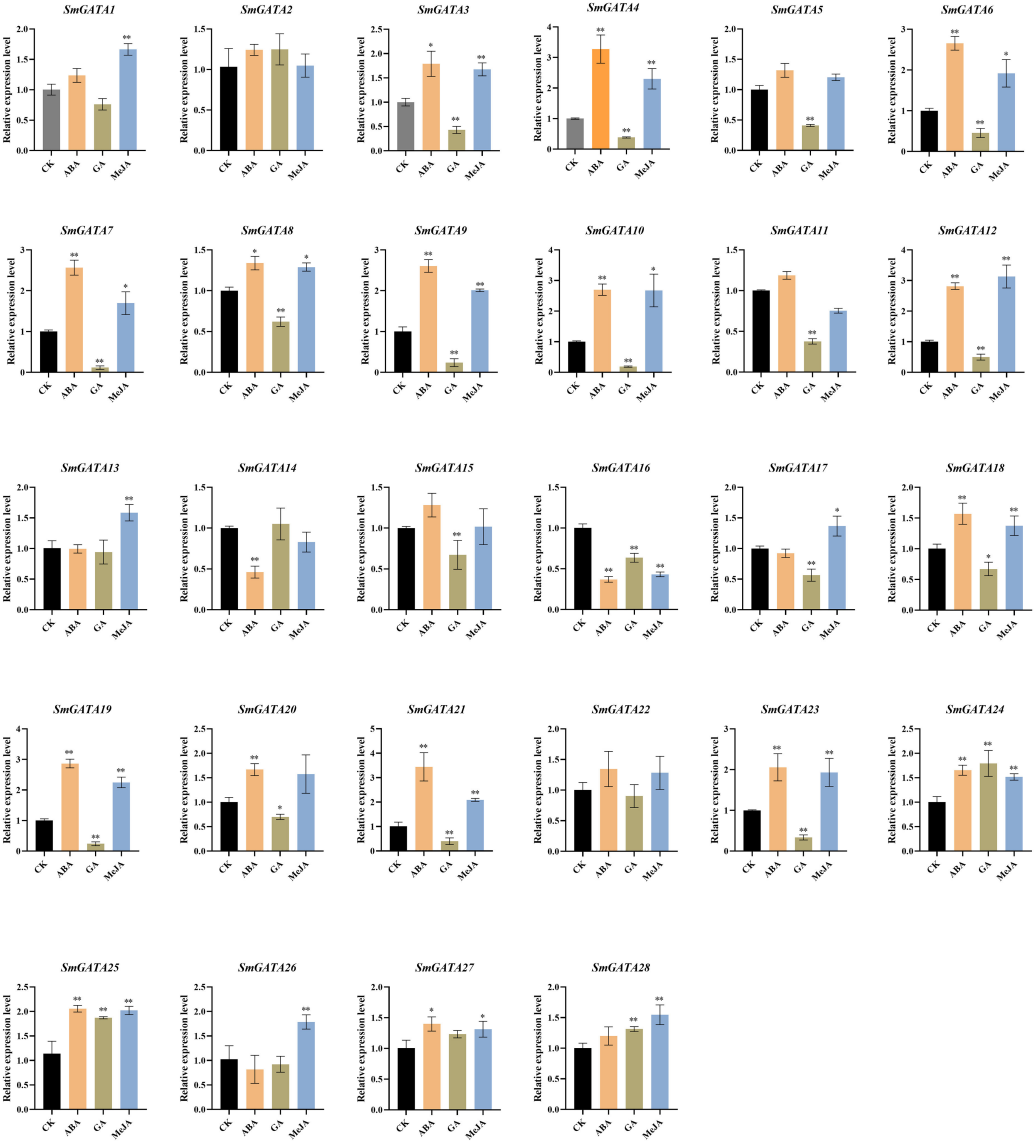
Expression profiles of eggplant *GATA* genes in response to ABA, GA, and MeJA treatment. *SmActin* was used as the internal reference control to normalize template levels. Relative mRNA levels are represented as the mean ± SD (n=3). Statistically significant differences were assessed using Student’s *t*-test (*P < 0.05, **P < 0.01).

The hormone signals and environmental changes are critical to fruit development and ripening (Santner et al., 2009; Leng et al., 2014; Kuhn et al., 2020; Guo et al., 2021). In our study, the analysis of *cis*-acting elements in the 2000 bp sequence upstream of the *SmGATAs* promoter revealed that most members of this family respond to avariety of hormones and stresses, indicating that SmGATAs may be regulated by light, stresses, and phytohormones (**Figure 3**). In our study, we measured the expression levels of *SmGATA* genes in response to ABA, GA, and MeJA treatment by RT-qPCR (**Figure 7**). As expected, the *SmGATA* genes showed diverse expression patterns during treatment with different hormones. During ABA treatment, 16 *SmGATA* genes were significantly upregulated, indicating that these genes positively respond to ABA treatment. A majority of *SmGATA* genes were significantly downregulated under GA treatment, The 28 *SmGATA* genes responded to MeJA to varying degrees. In general, most *SmGATA* genes were sensitive to different hormone treatments. It is worth noting that 12 *SmGATA* (*SmGATA3*, *4*, *6*, *7*, *8*, *9*, *10*, *12*, *18*, *19*, *21* and *23*) exhibit similar expression trends under three treatments. Furthermore, 7 *SmGATA* genes (*SmGATA4*, *9*, *10*, *12*, *19*, *21* and *23*) were significantly upregulated by more than two times under ABA and MeJA treatments, and downregulated by more than two times under GA treatment. Taken together, the response of most *SmGATA* genes to ABA and MeJA signaling were consistent, and the expression level showed an upward trend. but it exhibited contrary trend of the GA signaling pathway. Notably, three hormones treatment (ABA, GA, and MeJA) had no significant effect on the expression of *SmGATA2*.

### 3.9 Co-expression of network analysis of *SmGATA* with anthocyanin biosynthesis genes, light signaling genes and hormone signals genes

**Figure 8:**
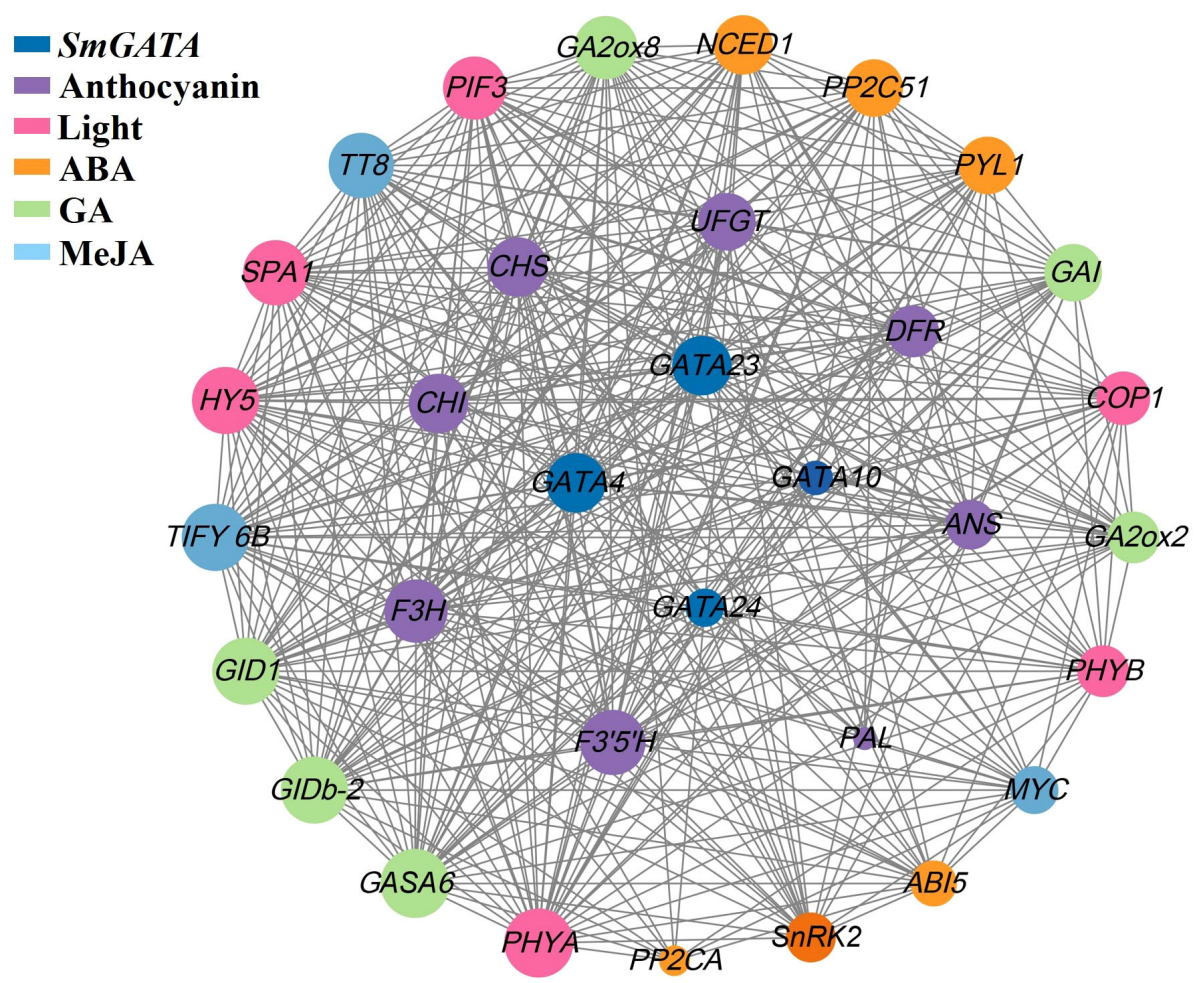
Co-expression network analysis of *GATA*, anthocyanin biosynthesis, light signaling, and multiple hormone signaling genes in eggplant.

We assessed the transcriptome data of eggplant fruits with different colors to examine the interactions between *GATA* genes, light-responsive genes, multiple hormone signaling genes, and anthocyanin biosynthesis genes in eggplant fruits. Four *SmGATA* genes (*SmGATA4*, 10, 23, and 24) according to comprehensive analysis of the expression patterns of *SmGATA* genes under different developmental stages, as well as under light and hormone treatments were screened. We analyzed the expression of 8 anthocyanin biosynthesis genes (*PAL*, *CHS*, *CHI*, *F3H*, *F3′H*, *DFR*, *ANS* and *UFGT*) and 6 light-responsive genes to identify genes that might regulate anthocyanin biosynthesis during fruit development. As shown in **Figure 8**, genes encoding light signaling components (*HY5*, *PIF3*) and anthocyanin biosynthesis genes were co-expressed with ABA, GA and MeJA signaling pathway genes, suggesting that these three hormones might play major roles in anthocyanin biosynthesis in eggplant. Furthermore, the expression patterns of four *SmGATA* genes (*SmGATA4, 10, 23*, and *24*) were correlated with the expression patterns of anthocyanin biosynthesis genes and light-responsive genes. Notably, *GATA4* and *GATA23* expression levels were highly correlated with anthocyanin biosynthesis and light-responsive gene expression, and these genes were co-expressed with genes encoding components of the ABA, BR, and MeJA signaling pathways. These results showed that *GATA* genes likely play major roles in regulating anthocyanin biosynthesis by integrating the light, ABA, GA, and MeJA signaling pathways.

### 3.10 Subcellular localization of eggplant GATA proteins

**Figure 9:**
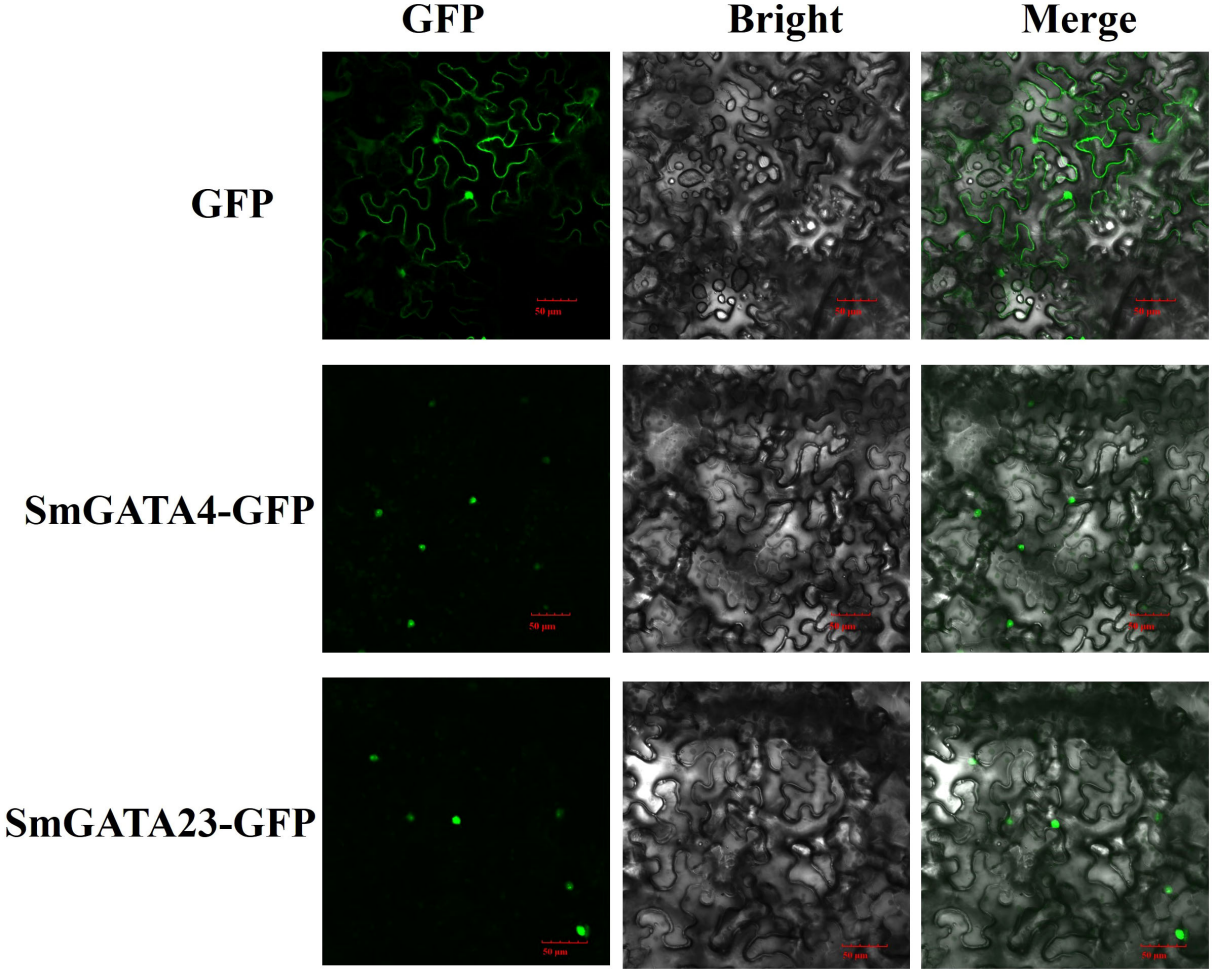
Subcellular localization of two GFP-fused SmGATA proteins. The two SmGATA-GFP fusion proteins (SmGATA4-GFP and SmGATA23-GFP) were transiently expressed in *N. benthamiana* leaves and observed by fluorescence microscopy 48 h later. Bar = 50 μm.

Transcription factors play many regulatory roles in plants. The nuclear localization of transcription factors is important for their regulatory roles. Most GATA proteins are located in the nucleus, such as AtGATA2 (Luo et al., 2010), BdGATA13 (Guo et al., 2021). To examine the subcellular locations of GATA proteins in eggplant, we transiently transformed *Nicotiana benthamiana* epidermal cells with two *SmGATA* genes (*SmGATA4* and *SmGATA23*) and examined the subcellular localization of the resulting GFP-tagged fusion proteins. As shown in **Figure 9**, SmGATA4-GFP and SmGATA23-GFP showed green fluorescent signals in the nuclei of *N. benthamiana* epidermal cells. These results revealed that SmGATA4 and SmGATA23 were nuclear proteins, which was consistent with previous results and their presumed roles as transcription factors (Luo et al., 2010; Guo et al., 2021).

## 4 Discussion

### 4.1 Evolutionary analysis of eggplant *GATA* genes

In the current study, we identified 28 *GATA* genes in the genome database of eggplant. The number of *GATA* genes varies among plant species; for example, there are 30 *GATA* family members in *Arabidopsis* (Reyes et al., 2004), 28 in rice (Reyes et al., 2004), 30 in tomato (Yuan et al., 2018) and 35 in apple (Chen et al., 2017). These discrepancies may arise from variations in genome size and complexity among these species. The 28 SmGATAs were divided into four subfamilies (I, II, III and IV) (**Figure 1**), which is consistent with most GATA family studies (Du et al., 2022; Feng et al., 2022), indicating that the GATA family is relatively stable during evolution. We found that the amino acid motifs and gene structures of *SmGATA* genes are relatively conserved (**Figure 2B**), the 28 SmGATAs contain 1-10 motifs, with motif 1 being widely distributed, suggesting its role as the Zinc finger GATA motif in this family. At the same time, we found that the *SmGATA*s genes of subfamily III and IV are rich in CDS (**Figure 2C**). The distribution of conserved motifs and gene structures is similar among members within the same subfamily, but there are significant differences between subfamilies, which is consistent with prior research findings (Manfield et al., 2007; Yu et al., 2019). Currently, a large number of *cis*-acting elements and their associated functions or pathways have been deciphered. By analyzing the promoter sequences of *SmGATA* genes in eggplant, a remarkable number of hormone and stress response elements have been identified ((**Figure 3**), indicating that they may be involved in multiple biological processes. As shown in **Figure 4**, the 28 *SmGATA* genes exhibit uneven distribution across eggplant chromosomes, which may be referred to as the differences in the size and structure of the chromosomes. Notably, 15 pairs of SmGATAs in the eggplant genome were duplicated, suggesting gene duplication as a contributing factor to the expansion of the *GATA* gene family in eggplant.

### 4.2 Potential roles of *SmGATA* genes in eggplant fruit development and ripening

The development and ripening of eggplant directly afect the quality of fresh fruit, and which is a complex physiological and biochemical process that is influenced by various transcription factors and regulatory proteins (Li et al., 2021b; Yang et al., 2022; Li et al., 2024). our results revealed that some *SmGATA* genes were highly expressed in late stage of fruit developments (**Figure 5**), implying potential roles in development and fruit ripening. There are previously reported examples of *GATA* genes being involved in these processes in *A. thaliana*, where GATA proteins have been found to be involved in chlorophyll synthesis and foral development (Bi et al., 2005). In chrysanthemum, CmGATA4 can directly bind to the key gene *CmCCD4a-5* for carotenoid degradation, acts as a negative regulator to lower the expression of CmCCD4a-5 resulting in carotenoid accumulation in the mutant (Huang et al., 2022). Additionally, in tomato, SlGATA17 protein interacts with SlHY5, and SlHY5 plays a role upstream of SlGATA17, which inhibits *SlGATA17* gene expression by binding to its promter (Wang et al., 2023). Furthermore, SlHY5 regulates fruit ripening both at the transcriptional level by targeting crucial genes involved in anthocyanin biosynthesis and ethylene biosynthesis, which also at the translational level by affecting the protein translation machinery (Wang et al., 2021). Here, the expression level of *SmGATA4*, *SmGATA23* and *SmGATA2*4 are significantly upregulated during fruit development (**Figure 5**), which revealed that these three genes are involved in the ripening process in eggplant. Finally, two genes (*SmGATA1* and *SmGATA26*) were downregulated during late fruit development, which means that these genes encode negative regulators of fruit ripening in eggplant.

### 4.3 Potential roles of *SmGATA* genes in response to light and multiple hormones

Light is an important factor that influences plant growth and development (Jaakola, 2013). Response promoter elements, G-box and GATA motif, is critical for promoter activation in response to the signals from multiple photoreceptors (Chattopadhyay et al., 1998). In fungi, such as *Neurospora*, two GATA-type factors bind to GATA element and regulate gene expression in response to light signal (Scazzocchio, 2000). Additionally, *In A. thaliana*, *AtGATA2* was reported as a rositive regulator of photomorphogenesis and GATA21 and GATA22 are induced by red light in a PIF3-dependent manner (Monte et al., 2004; Luo et al., 2010). Furthermore, anthocyanin accumulation is highly dependent on light in photosensitive eggplants. Based on our present results, three *SmGATA* genes are upregulated and two *SmGATA* genes are downregulated during light exposure, proposed that these differentially expressed genes might function in light-induced anthocyanin biosynthesis in eggplant (**Figure 6**).

Exogenous hormone treatment can promote fruit ripening (Forlani et al., 2019). As a non-climacteric fruit, mutiple hormones play an important role in regulating the ripening process of eggplants, including MeJA (Li et al., 2024). Additionally, the roles of SmGATA proteins in hormone signaling pathways are currently unclear. Several reports document the roles of *GATA* genes in hormonal pathways in other plants (Wei et al., 2023). TaGATA1–TaELF6-A1–TaABI5, which contributes to seed dormancy through the ABA signaling pathway in *Triticum aestivum* (Wei et al., 2023). The GATA-type transcription factors GNC and GNL/CGA1, is a negative regulatory factor of GA signaling, functions in downstream from DELLA proteins (Richter et al., 2010). Other example suggested GATAs can interact with JAZ, a key regulator in the jasmonic acid (JA) signaling pathway (Sen et al., 2016). In our study, RT-qPCR revealed that the *SmGATA* genes were responsive to numerous hormonal treatments (**Figure 7**). More than half of the *SmGATA* genes were regulated by more than one hormone treatment, providing that these genes may be involved in the interactions of different hormone signals at the physiological level. Furthermore, the expression patterns of *GATA* genes were highly correlated with those of anthocyanin biosynthesis genes, light-responsive genes, and ABA, GA and MeJA signaling pathway genes (**Figure 8**). In addition, *SmGATA4* and *SmGATA23* responded to three hormone signals and light treatment, indicating that these genes encode proteins that integrate light and hormone signals to regulate anthocyanin biosynthesis.

## 5 Conclusions

In this study, 28 *SmGATA* genes were identified in the eggplant genome database, and their gene structures and expression patterns were comprehensively investigated. The results illustrated that the *SmGATA* genes significantly contributed to fruit development and ripening, primarily manifested in the expression patterns of the *SmGATA* gene under light exposure and changes in hormone levels. Especially by the correlation between several *SmGATA* genes and the expression of light-responsive genes, various hormone signaling genes, and anthocyanin biosynthesis genes. Particularly noteworthy was the upregulation of several *SmGATA* genes in response to hormone treatments such as ABA, GA and MeJA, indicating their role in integrating light, ABA, GA and MeJA signaling pathways to regulate anthocyanin biosynthesis. In conclusion, our comprehensive analysis of the *SmGATA* family across the genome provides a basis for further investigations into the biological roles of these genes in fruit development and ripening.

## Data availability statement

The original contributions presented in the study are included in the article/**Supplementary Material**. Further inquiries can be directed to the corresponding authors.

## Author contributions

YW: Writing – original draft, Writing – review & editing. XL: Investigation, Writing – original draft. YM: Software, Writing – original draft. CJ: Investigation, Writing – original draft. YZ: Data curation, Writing – original draft. JH: Methodology, Writing – original draft. YLZ: Methodology, Writing – original draft. JL: Conceptualization, Methodology, Software, Writing – review & editing. KZ: Conceptualization, Funding acquisition, Writing – review & editing. ZL: Conceptualization, Software, Writing – review & editing.
