# Supplementary material for: Identification and expression profiling of SmGATA genes family involved in response to light and phytohormones in eggplant
Source: Front Plant Sci. 2024 May 28;15:1415921. doi: 10.3389/fpls.2024.1415921 (PMC11165305; doi:10.3389/fpls.2024.1415921)
Supplement: Supplementary file 1 [file DataSheet_1.zip › Data Availability Guidelines for Editors and Reviewers.pdf]

frontiers

Data Availability Checks

Guidelines for editors and reviewers

Table of Contents

1 Introduction 3
1.1 Data Availability Check at Frontiers 3
1.2 Your Assessment 3

2 Requirements For Authors 4
2.1 Data Availability Statements 4
2.2 Required Data Types 5
2.3 Recommended Repositories 8

3 Data Availability Indicator 9
3.1 How AIRA interprets Data Availability 9
3.2 Resolving the AIRA Indicator 10

4 Further Information 10
4.1 Chinese Data Restrictions 10
4.2 Sequencing by third parties 11

5 Ethics and Data Availability 11
5.1 Case Reports and/or Identifiable Data 11

1 Introduction

1.1 Data Availability Check at Frontiers

Frontiers is committed to open science and open data; we request that authors make available all data relevant to the conclusions of the manuscript. Generated data should be publicly available and cited in accordance with our 'Data citation guidelines'. Our policies on data availability are informed by community-driven standards, which Frontiers endorses, such as the Transparency and Openness (TOP) guidelines and the joint declaration of data citation principles produced by FORCE11.

1.2 Your Assessment

In order to assist Frontiers in upholding the aforementioned standards. Please consider whether the manuscript adheres to our requirements during your initial assessment. Please consider the following:

- Does the manuscript contain a required data type?
- Have the authors provided a suitable data availability statement?
- If the manuscript does contain required data, is it available in a public, community-supported repository?

Please note that a dedicated team within the Frontiers Research Integrity Department are contactable for any concerns you may find within a manuscript. Please email at: datapolicy@frontiersin.org.

For any further information on the requirements of authors, the guidelines Frontiers operates in and the requirements for required data deposit, please see the relevant section below.

2 Requirements For Authors

Authors are encouraged to make all materials used to conduct their research available to other researchers. Research materials necessary to enable the reproduction of an experiment should be clearly indicated in the Materials and Methods section. Relevant materials such as protocols, analytic methods, and study material should preferably be uploaded to an online repository providing a global persistent link/identifier. If this is not possible, authors are strongly encouraged to make this material available upon request to interested researchers, and this should be stated in the manuscript.

Frontiers requires that authors make the "minimal data set" underlying the findings described and used to reach the conclusions of the manuscript, available to any qualified researcher. The data should be FAIR – findable, accessible, interoperable, and reusable – so that other researchers can locate and use the data. However, exceptions are granted if data cannot be made publicly available for legal or ethical reasons. To comply with best practice in their field of research, authors are required to make certain types of data available to readers at the time of publication in public, stable, community-supported repositories.

2.1 Data Availability Statements

Data availability statements are required for all articles published with Frontiers. During the submission process, authors will be asked to detail the location of the raw data underlying the conclusions made in the manuscript, and whether it will be made available to other researchers following publication. Authors will also be asked for the details of any existing datasets that have been analysed in the manuscript. These datasets should be cited in accordance with our data citation guidelines.

Example of Acceptable Statement	Description of Correct Use
Original datasets are available in a publicly accessible repository:	
The original contributions presented in the study are publicly available. This data can be found here: [link/accession number].	Used for data that the authors have generated for their manuscript. Ideally authors should know to choose this for any paper with a required data type not protected by ethics/privacy concerns.
Existing datasets are available in a publicly accessible repository:	
Publicly available datasets were analysed in this study. This data can be found here: [link/accession number].	Used for data that was already publicly available prior to the authors starting their paper (authors did not create data for their MS), and only 1/few datasets used. Authors should include links or unique identifiers to the specific datasets, not just the name of the repository.
Information for existing publicly accessible datasets is contained within the article.	These two are very similar, used for data that was already publicly available prior to the authors

The datasets presented in this study can be found in online repositories. The names of the repository/repositories and accession number(s) can be found in the article/supplementary material.	starting they paper (authors did not create data for their MS), and a large number of datasets are used.
All relevant data is contained within the article:	
The original contributions presented in the study are included in the article/supplementary material, further inquiries can be directed to the corresponding author/s	This is for non-required data types where we still push authors to include information, this is also for 'false flags' such as Sanger Sequencing or PCR where the only required data is a list of primers within the article.
Restrictions or ethical concerns apply to the datasets:	
The datasets presented in this article are not readily available because [VALID REASON]. Requests to access the datasets should be directed to [text input].	This is for required data types that are protected. This can occur for a number of reasons but is most likely to do with ethics or other privacy concerns.
The datasets for this article are not publicly available due to concerns regarding participant/patient anonymity. Requests to access the datasets should be directed to the corresponding author.	This is for Case Report manuscripts or other manuscripts that include required data that cannot be shared for ethical concerns.
Data has been obtained from a third-party:	
The data analyzed in this study was obtained from [SOURCE], the following licenses/restrictions apply [RESTRICTIONS]. Requests to access these datasets should be directed to [NAME, EMAIL].	This is for required data that the authors outsourced to a third-party company or that the authors have sourced after undergoing specific training and/or permissions to access.
Datasets are available on request:	
The raw data supporting the conclusions of this article will be made available by the authors, without undue reservation.	This is for non-required data types that do not require further information in the MS or supp material.

2.2 Required Data Types

<u>Genetic and genomic sequence (DNA/RNA)</u>

- Genetic sequencing is used to determine the base nucleotide sequence of a gene/genes.
- Genomic sequencing is the same principle, but for almost all the DNA in an organism.
- There are many ways to sequence genomic data such as:
  - DNA sequencing
  - RNA sequencing
  - Methylation Sequencing
  - High-Throughput Sequencing
  - Long-Read Sequencing
- An example manuscript that uses this data can be found at: https://www.frontiersin.org/articles/10.3389/fgene.2023.1244493/full#h7

Metagenomic sequence

- Similar to genomic sequencing but can look into multiple organisms in a given sample at the same time.
    - For example, looking at all the bacteria in a complex sample.
- Happens in two main ways:
    - Amplicon sequencing (often through the 16s rRNA gene, hence its other name - 16s rRNA sequencing)
    - Shotgun sequencing
- An example manuscript that uses this data can be found at: https://www.frontiersin.org/articles/10.3389/fcimb.2023.991011/full#h7

DNA and RNA trace or short-read sequencing data

- Short-read sequencing is very similar to long-read sequencing but produces a shorter output.
- The DNA or RNA are first split into smaller sections, and these smaller sections are then sequenced individually in a very similar way to long read sequencing.
- 'Trace' is another name for the smaller sections that the genetic data is broken down into.
- An example manuscript that uses this data can be found at: https://www.frontiersin.org/articles/10.3389/fpls.2021.657240/full#h6

Genetic polymorphism data, including SNP and CNV data

- Polymorphic genes are defined as 'two or more variants of the same gene that exist within the same population' (such as hair colour).
- Polymorphic genes become distinct from genetic mutations when they occur in at least 1% of a given population.
- There are many types of genetic polymorphisms:
    - SNPs are single nucleotide changes within DNA and are the most common form of genetic variation.
    - CNVs are areas of repeats within DNA, where the number of times the sequence repeats, varies from person to person.
    - Other types include 'indels' (insertion/deletion of nucleotides in a sequence), transposable elements (sections of DNA that can move their location within a genome) and microsatellites.
- An example manuscript that uses this data can be found at: https://www.frontiersin.org/articles/10.3389/fimmu.2022.842745/full#h6

Gene expression data; chromatin immunoprecipitation data (deep-sequencing or microarray)

- Gene expression data focuses on the process by which the information stored in genes is transcribed into a functional gene product.
- Most gene expression data is collated by measuring mRNA levels, although it can also be done by directly measuring protein levels.
- Interactions between genes and their functional products are chemically identified before samples are sequenced to convert the interactions into a quantitative value of gene expression.
- An example manuscript that uses this data can be found at: https://www.frontiersin.org/articles/10.3389/fimmu.2021.682094/full#h6

Data linking genotype to phenotype

- A genotype is the sequence of nucleotides that make up a gene, a phenotype is the physical representation of that as sequence.

- Studies looking into how genotypes link and convert into the observed phenotypes include Genome-Wide Association Studies (GWAS)
- An example manuscript that uses this data can be found at: https://www.frontiersin.org/articles/10.3389/fgene.2021.643883/full#h7

Protein sequence data

- The primary amino acid sequence of a protein determines its structure and its function.
- The most common way this is done is through mass spectroscopy (however Edman degradation is another method)
- After samples are separated through liquid chromatography, the individual qualities of the separated samples can be determined and used to identify the amino acids when compared to known standards.
- An example manuscript that uses this data can be found at: https://www.frontiersin.org/articles/10.3389/fnut.2023.1144346/full#h6

Proteome profiling data

- A proteome is all the proteins and their sequences in a given sample, this could be a whole organism, a group of organs, or any biologically defined system.
- Similarly to how metagenomics looked at multiple genetic sequences, proteomics looks at multiple protein sequences.
- An example manuscript that uses this data can be found at: https://www.frontiersin.org/articles/10.3389/fphys.2023.1150521/full#h4

Small molecule, protein, protein complex data structural data

- Proteins don't only exist as their amino acid chain but also have 3D structures and angles that the amino acids are held in for function.
- Protein complexes occur when at least two polypeptide chains are associated, in these cases the relationship between the two chains can also be measured.
- As well as proteins, the crystalline structure of small molecules also needs to be deposited.
- There are many ways that this can be measured, common ones include X-ray crystallography/ diffraction and Nuclear Magnetic Resonance
- An example manuscript that uses this data can be found at: https://www.frontiersin.org/articles/10.3389/fmolb.2022.960248/full#h7

Taxonomy data

- Taxonomy is the branch of science that deals with naming organisms.
- When a potentially new organism is discovered, it is compared against all known taxonomy data to see if it has already been identified and named, if not, then it is a novel organism and requires deposit in a suitable database.
- An example manuscript that uses this data can be found at: https://www.frontiersin.org/articles/10.3389/fmars.2019.00381/full (note that the zoobank ID is within the abstract, not the Data Availability Statement)

2.3 Recommended Repositories

A data repository is a data archive where large datasets can be stored, managed, and accessed directly linked to a specific project or article, granting the storage of all the required data within the same location.

Authors are required to deposit their data in public, community-supported repositories. The importance of community-supported repositories lies on the criteria for anonymous peer-review, data access, preservation, and resource stability.

Community-supported repositories require the data to be deposited in a specific format including metadata. If community repositories were not available, generalist or institutional repositories could be used. However, they do not hold strict requirements on data format and usually do not request metadata.

Below, you can find the recommended repositories for each mandatory data type.

Data Type	Recommended Repository
Genetic and genomic sequence (DNA/RNA)	- Genbank<br>- DNA Data Bank of Japan (DDBJ)<br>- European Nucleotide Archive (ENA)<br>- National Genomics Data Centre<br>- Sequence archive element of Chinese National Genebank (CNSA)
Metagenomic sequence	- EBI Metagenomics
DNA and RNA trace or short-read sequencing data	- NCBI Sequence Read Archive (SRA)
Genetic polymorphism data, including SNP and CNV data	- dbSNP<br>- dbVar<br>- European Variation Archive
Gene expression data; chromatin immunoprecipitation data (deep-sequencing or microarray)	- ArrayExpress<br>- Gene Expression Omnibus (GEO)
Data linking genotype to phenotype	- dbGaP
Protein sequence data	- UniProt
Proteome profiling data	- PRIDE<br>- PeptideAtlas<br>- ProteomeXchange
Protein-protein interaction data	- Database of Interacting Proteins (DIP)
Small molecule, protein, protein complex data, structural data	- Crystallography Open Database<br>- Cambridge Structural Database (CSD)<br>- wwPDB (Protein DataBank)<br>- Electron Microscopy Databank (EMDB)<br>- MetaboLights<br>- Human Metabolome Database<br>- PubChem
Taxonomy data	- Zoobank
Plant data	- e!DAL
Trait data (plant trait)	- TRY database

Agriculture data	- The National Agricultural Biotechnology Information Center (Korean)
COVID data hub	- GISAID
Microbiology (Chinese)	- National Microbiology Data Center (NMDC)
General data	- Zenodo
Flow cytometry data	- Flow Repository
Brain imaging data; neuroimaging data	- OpenNeuro<br>- iNDI<br>- NITRC<br>- NeuroVault [Statistical maps]
Phenology data	- National Phenology Network
Enzymology and biocatalysis data	- STRENDA database

Exceptions: If the authors have failed to deposit their data in one of the recommended repositories, we encourage the use of more general repositories, such as FigShare, Dryad Digital Repository or Github. These cases should be exceptional as the recommended repositories are preferred due to their specificity to each data type.

Note: If you are directed to the Worldwide Protein Data Bank, please note that this is a "mother site" for the region or type specific PDBs. You cannot search accessions from here. Scroll down to the section entitled "wwPDB Members" to find the relevant repository, e.g. https://www.ebi.ac.uk/pdbe/ for the European PDB.

3 Data Availability Indicator

3.1 How AIRA interprets Data Availability

The data availability indicator can take a number of different formats depending on the information provided within the manuscript and by the authors at submission. Below you will find the potential indicator formats:

Keyword detection and authors provide correct statement.

This indicator will be orange. Please check the type of data mentioned in the paper (and the statement provided by the authors) to ensure that it is a mandatory data type, and that it has been deposited successfully. If it has, then the indicator can be resolved as 'data availability is OK'. If it hasn't, please consider reminding the authors of their responsibility to deposit, and release, the data.

Non keyword detection but author chose statement that naturally puts it in blocked.

This indicator will be orange. Please check the methodology to ensure that a mandatory data type is not mentioned. If this is the case, the authors have accidentally chosen an incorrect statement. No action is required, and our Production Team will correct this in due course. If a mandatory data type is mentioned and AIRA has ruled incorrectly, please consider reminding the authors of their responsibility to deposit, and release, the data.

Keyword detection and authors provide incorrect statement.

This indicator will be orange and it suggests that the authors have got a mandatory data type and have not provided a correct statement for deposit. Please check the methodology and consider following up for deposit, if required. If there is no mandatory data, no action is required, and our Production team will correct this in due course.

Non keyword and authors chose a statement that doesn't need checking.

This indicator will be green and requires no further action unless something in the manuscript indicates that the AIRA solution is incorrect.

3.2 Resolving the AIRA Indicator

Below please find the three options for resolving the AIRA Data Availability indicator.

Data availability is OK.

This is for when the information provided by the authors matches the data type in the paper. This is the correct indicator for when the authors provide a Data Availability Statement showing deposit that is already publicly available. It is also the correct indicator for when AIRA has falsely flagged a data type by keyword - providing the statement given by the authors is correct.

Data archived, awaiting release upon acceptance.

This is for when authors have been able to confirm that a repository has their data, but the data is still currently private. This is very rarely the initial solution to the data indicator, normally it follows after a request for data and the authors have provided proof of deposit but wish to keep it private until acceptance.

Data to be publicly archived during review.

This is for when authors need to deposit and there is no indication that it has been done prior to submission. Resolve to this indicator and consider reminding the authors of their responsibility to deposit, and release, mandatory data types.

4 Further Information

4.1 Chinese Data Restrictions

Chinese authors of manuscripts with 'required' data types may say that they cannot make their data public due to national restrictions in China. It is true that authors could lose their job or be sent to prison if they do not follow these restrictions. However, they can still deposit their data in the China National Genebank (CNGB). Please contact the data team at the following address to raise this: datapolicy@frontiersin.org

4.2 Sequencing by third parties

Some authors can outsource sequencing to a third-party company and then use this to say they cannot deposit as they do not have the raw files. In these cases, Frontiers need to request that the authors contact the company and obtain their raw files. Please contact the data team at the following address to raise this: datapolicy@frontiersin.org.

5 Ethics and Data Availability

We strongly encourage sharing the maximal amount of data; however, where ethical, legal or privacy issues are present the data should not be shared. In cases where some or all data cannot be shared for legal, ethical or privacy restrictions, the authors should make these limitations clear in the Data Availability Statement at the time of submission.

Possible limitations to making data publicly available include patient confidentiality and participant privacy. Authors should ensure that the data shared are in accordance with the ethical consent provided by participants on the use of confidential/identifiable human data. We require that the authors demonstrate that publication of such data does not compromise the anonymity of the participants or breach local data protection laws.

In situations where access is restricted to protect confidential or proprietary information, authors are required to explain the restrictions on the dataset and make the data available upon request with permission of the third party. The Data Availability Statement should include all necessary contact information to request access to the dataset.

For any concerns, please contact the data team at: datapolicy@frontiersin.org

5.1 Case Reports and/or Identifiable Data

We do not encourage data deposition for manuscripts that include directly/indirectly identifiable data. For Case Report manuscripts, or any manuscript that includes directly/indirectly identifiable data, please do not request data deposition.

If the authors have already provided a data accession number or link to the data, that is OK - but otherwise, please do not request data deposition.
