# Supplementary material for: Identification and expression profiling of SmGATA genes family involved in response to light and phytohormones in eggplant
Source: Front Plant Sci. 2024 May 28;15:1415921. doi: 10.3389/fpls.2024.1415921 (PMC11165305; doi:10.3389/fpls.2024.1415921)
Supplement: Supplementary file 1 [file DataSheet_1.zip › Figure S1.DOC]

**Figure.S1**: Chromosome distribution of eggplant *GATA* genes. The scale on the left is in megabases (Mb).
