# Supplementary material for: Identification and expression profiling of SmGATA genes family involved in response to light and phytohormones in eggplant
Source: Front Plant Sci. 2024 May 28;15:1415921. doi: 10.3389/fpls.2024.1415921 (PMC11165305; doi:10.3389/fpls.2024.1415921)
Supplement: Supplementary file 1 [file DataSheet_1.zip › Figure S2.DOC]

**Figure. S2**. Multiple sequence alignments of the conserved domains of the SmGATAs and webLogos showing the conserved domains in SmGATA proteins. A, Multiple sequence alignments of the conserved domains are shown. The sequences were aligned using DNAMAN6.0 and BioEdit. B, WebLogos showing the conserved domains in SmGATA proteins. The x-axis indicates the conserved sequences of the domain. The height of each letter indicates the conservation of each residue across all proteins. The y-axis shows a scale of the relative entropy, which reflects the conservation rate of each amino acid.
