# Supplementary material for: Identification and expression profiling of SmGATA genes family involved in response to light and phytohormones in eggplant
Source: Front Plant Sci. 2024 May 28;15:1415921. doi: 10.3389/fpls.2024.1415921 (PMC11165305; doi:10.3389/fpls.2024.1415921)
Supplement: Supplementary file 1 [file DataSheet_1.zip › Table S1.DOC]

**Table S1. Primer sequences used for RT-qPCR**

| **Genes** | | **Forward Primer (5’to 3’)** | | **Reverse Primer (5’to 3’)** |
| --- | --- | --- | --- | --- |
| *SmGATA1* | | CCTGCCGGAAGTTTGAAGG | | CTGAAACAGTCGACGTCGGA |
| *SmGATA2* | | CATCCTGAACCTACACTAAG | | GGCTCGCTTTTGTCAGTGGG |
| *SmGATA3* | | GGAGATCTGGTCCTTCTGG | | CTTTGCTTAGCTTCCCATC |
| *SmGATA4* | | CGAAAACACTCTGCAATGCC | | CCATAACTTTCCGGTGAG |
| *SmGATA5* | | ATGGAGATGGATCTAAGTGATAAGG | | CTCATCGGCTTCTTATGTCC |
| *SmGATA6* | | CAAGCCTTCCCCACCACAGAAT | | GAGAGATTTTTCCGGTAGG |
| *SmGATA7* | | CATCTTCGGTGGATTGTACTC | | AGTGCGACCTGACTTATGACC |
| *SmGATA8* | | GCCTGAGAGACCGTTGAAAATG | | CAACGAAGCCATTGGAGAAGTCA |
| *SmGATA9* | | CTCTATGCTCTTCTCCCATGAATA | | GGCACGAGTAGACTTGTATTGAGC |
| *SmGATA10* | | CTGGGTATTCGCTGATGACGGG | | AGGAAGAAGCAGAGGAAGTTGAAGA |
| *SmGATA11* | | TCTGCTGATTCTTCCACCGTTACC | | GTTTGCTAAAGGGATGAACTGAAGG |
| *SmGATA12* | | GATGATCTTCTCGATTTCTCCAATG | | CTACATCATCACTAGGAAGGCAGAG |
| *SmGATA13* | | GGATGATGGAGGGGAAGAGACTATG | | CACAGCAGTAGGAATGTCACATC |
| *SmGATA14* | | CATAGTCGTTCTTTTCCTGTTCGC | | GTACTGAATCTGGGCTGTGATGATC |
| *SmGATA15* | | AATCAAAGAAATCAGCGGCACTTG | | GTCCTTTCCCATTTTCATTCCTGTC |
| *SmGATA16* | | TCCTCGTCCAACAACTTTTAATCCT | | GTTGTGCCACTGGATTCTGATTTG |
| *SmGATA17* | | CAGCAGCAGTGGAGAAAGTACGA | | TGCTTGTTCTTCGTCACCCAAC |
| *SmGATA18* | | AAGTAGCAATGAGGAGAAGGAAC | | GGAGCAAAATTAGATGTTGGTAGG |
| *SmGATA19* | | GTCCTATCAAAGTCCAATTTCTGTC | | GTTGTCTGTAGATGTTGTATTAGG |
| *SmGATA20* | | GAGCAGAACCTTGTGACTTTGAGG | | AGTATCTTCATCTAAACCCTTACGG |
| *SmGATA21* | | AATTCCACTGATTGTTCCACCGTC | | GATTCTTCCACAAAATTCGATAGCC |
| *SmGATA22* | | GCTGAACCAGATGATCTTGATGAT | | AGAGCCAGAACTTGACCTATTGC |
| *SmGATA23* | | GGATCAAGTACAATAAGAAGAGGAG | | TCAATAGTATCGCCGCTTGTTC |
| *SmGATA24* | GCTGTGTTAACTTAAAAGTACCGGT | | GTCTTATCAGCTCCACAGTGTTGG | |
| *SmGATA25* | TTCAATCCCCGGCCAGTAAATCA | | CGCAGGCTGGTGGTTATTATCCT | |
| *SmGATA26* | GAAACTAGAAGATCAAAAGCAGCA | | GAAATTTGTCCCATTATTTGTTGC | |
| *SmGATA27* | ATGTACAGAAGAGAAACAATGACCG | | AGCCAATGTGATGAGAAGAGTCTT | |
| *SmGATA28* | ATCGACCAGCTGCTAGTCCTACATT | | GCTTCTCTCTTTCTTCCCATGATCA | |
| *SmActin* | TGGAATATGGAACAGACAAGATGG | | CAAGAAGCCTAATCGCAGCAG | |
