# Supplementary material for: Identification and expression profiling of SmGATA genes family involved in response to light and phytohormones in eggplant
Source: Front Plant Sci. 2024 May 28;15:1415921. doi: 10.3389/fpls.2024.1415921 (PMC11165305; doi:10.3389/fpls.2024.1415921)
Supplement: Supplementary file 1 [file DataSheet_1.zip › Table S2.DOC]

**Table S2.** Abbreviations of differentially expressed genes

| **Type** | **Gene ID** | **Gene Name** | **Abbreviation** |
| --- | --- | --- | --- |
| Anthocyanin Biosynthesis | SMEL_005g227920.1 | Phenylalanine ammonia lyase | *PAL* |
|  | SMEL_005g227980.1 | Chalcone synthase | *CHS* |
|  | SMEL_010g353630.1 | Chalcone isomerase | *CHI* |
|  | SMEL_010g347130.1 | Flavanone 3-hydroxylase | *F3H* |
|  | SMEL_012g398880.1 | Flavanone 3’-5’hydroxylase | *F3'5’H* |
|  | SMEL_001g136730.1 | Dihydroflavonol-4-reductase | *DFR* |
|  | SMEL_008g318060.1 | Anthocyanidin synthase | *ANS* |
|  | SMEL_005g234690.1 | UDP-glucose: flavonoid-3-O-glucosyltransferase | *UFGT* |
| Light Perception and Signaling | SMEL_004g227510.1 | Phytochrome A | *PHYA* |
|  | SMEL_001g141950.1 | Phytochrome B | *PHYB* |
|  | SMEL_011g363370.1 | Constitutive photomorphogenic 1 | *COP1* |
|  | SMEL_008g303770.1 | Elongated hypocotyl | *HY5* |
|  | SMEL_003g192690.1 | Phytochrome interacting factors 3 | *PIF3* |
|  | SMEL_010g353260.1 | Suppressor of PHYA 1 | *SPA1* |
| ABA Signaling | SMEL_008g299750.1 | 9-*cis*-epoxycarotenoid dioxygenase | *NCED* |
|  | SMEL_004g219530.1 | Abscisic acid-insensitive 5 | *ABI5* |
|  | SMEL_011g364700.1 | Sucrose non-fermenting-1-related protein kinase 2 | *SnRK2* |
|  | SMEL_006g251030.1 | Protein phosphatase 51 | *PP2C51* |
|  | SMEL_004g219590.1 | Protein phosphatase 2CA | *PP2CA* |
|  | SMEL_004g213050.1 | PYR1-like 1 | *PYL1* |
| MeJA Signaling | SMEL_001g151410.1 | TIF(F/Y)XG-6B | *TIFY-6B* |
|  | SMEL_001g128250.1 | v-myc avian myelocytomatosis viral oncogene homolog | *MYC* |
|  | SMEL_009g326640.1 | Transparent testa 8 | *TT8* |
| GA Signaling | SMEL_004g205560.1 | Gibberellin 2-oxidase 8 | *GA2_OX_8* |
|  | SMEL_001g136570.1 | Gibberellin 2-oxidase 2 | *GA2_OX_2* |
|  | Solanum_melongena_newGene_4439 | Gibberellic acid insensitive | *GAI* |
|  | SMEL_006g242410.1 | GA-insensitive dwarf 1 | *GID1* |
|  | SMEL_009g329080.1 | GA-insensitive dwarf b-2 | *GIDb-2* |
|  | SMEL_006g243560.1 | GA-Stimulated in Arabidopsis 6 | *GASA6* |
