# Supplementary material for: Identification and expression profiling of SmGATA genes family involved in response to light and phytohormones in eggplant
Source: Front Plant Sci. 2024 May 28;15:1415921. doi: 10.3389/fpls.2024.1415921 (PMC11165305; doi:10.3389/fpls.2024.1415921)
Supplement: Supplementary file 1 [file DataSheet_1.zip › Table S3.DOC]

**Table S3. Primer sequences used for gene amplification**

| **Genes** | **Forward Primer (5’to 3’)** | | | **Reverse Primer (5’to 3’)** | | |
| --- | --- | --- | --- | --- | --- | --- |
| *SmGATA4-*1302 | acgggggactcttgaccatggtaATGGATGTCTACGGTCTGC | | | tctcctttactagtcagatctacGCTGGCCCGAAAGTGATGT | | |
| *SmGATA23-*1302 | acgggggactcttgaccatggtaATGGTGGATATAAGTGATAAAGT | | | tctcctttactagtcagatctacAGCATAAACAGAACCACAAGA | | |
|  | |  |  | |  |  |
